# Design and Superior Performance of a New Endotracheal Tube to Avoid the Asphyxiation of Premature Infants

**DOI:** 10.7759/cureus.47655

**Published:** 2023-10-25

**Authors:** Juan N Walterspiel

**Affiliations:** 1 Pediatrics, Independent Medical Contractor, LocumTenens, Menlo Park, USA

**Keywords:** endotracheal tube tip elevation, endotracheal intubation, steering, esophageal intubation, epiglottis, controlled, prospective, design, premature neonates, endotracheal tube

## Abstract

Background

Neonatal endotracheal intubation attempts often fail, with failures typically attributable to unintended esophageal intubation, with asphyxia, brief or prolonged, as the consequence. Standard-of-care neonatal endotracheal tubes have changed little over recent decades, even as the gestational age of neonates thought eligible for resuscitation and intensive care has decreased.

Methods

A new neonatal endotracheal tube was patterned after the soft steering mechanism of a two-string fishing line trocar. The new tube remains patent throughout the intubation for air movement and CO_2_ detection and allows for a finger on the intubator's hand to stiffen, curve, and elevate the tip of the tube over the epiglottis and into the trachea without occluding the vision through a laryngoscope. This tube's engineering principles were studied prospectively in a controlled open-label pilot study in premature infants. Infants were observed during 12 intubations in a one-to-one comparison with standard practice.

Results

The new design in comparison to a conventional neonatal endotracheal tube (CNETT) was found to be superior. The average intubation time (mean 36.6 sec, median 30 sec) was shorter (mean 44.6 sec, median 45 sec) in the new design. Intubation attempts were fewer (0 vs. 3), and unintended esophageal intubations were also fewer (0 vs. 4).

Conclusion

Tracheal intubation of premature infants with the new soft-steering mechanism endotracheal tube was associated with less asphyxia, fewer intubation attempts, and fewer esophageal intubations.

## Introduction

Up to one-half of endotracheal intubations of premature infants are associated with unintended intubations of the esophagus [1,2]. Esophageal intubation asphyxiates the newborn, can cause intraventricular hemorrhage, and has detrimental long-term consequences [3-7]. Various approaches have been taken to mitigate this situation by training pediatric residents more effectively [8-11]. The mechanical devices and trocars currently used to facilitate neonatal intubation are proficiency-dependent and have long remained unchanged. They have low safety margins, can traumatize and even perforate delicate tissues, and trocars, if used, necessarily occlude the tube, delaying confirmation of placement and prolonging asphyxia. Further, trocars while being removed may dislodge the tube.

The status quo in perinatal endotracheal intubation is overdue for innovation.

## Materials and methods

Design targets for a new neonatal endotracheal tube (NNETT) were established by the author (JNW) to address the challenging issues of neonatal intubation. A new neonatal endotracheal tube (NNETT) was designed to meet four targets: fine-motor control mechanism for intubators to curve the tube tip to slide over the epiglottis; tube patency throughout the intubation allowing intubators to detect air movements from the lungs, indicating tracheal intubation at the earliest possible moment; elimination of a trocar or guidewire so as to minimize tissue trauma and render accidental perforation impossible, and to allow one-handed insertion of the endotracheal tube under visualization of the epiglottis and cords. Engineering solutions to meet those four requirements were developed, underwent testing and improvements by JNW, and were built to be evaluated in this unblinded controlled pilot study in preterm infants. The mechanical principles of the NNETT, a soft, fishing line trocar with a ring attached, had been shown to be superior to a wire trocar in prematurely delivered sheep at the University of Texas Southwestern Medical School Dallas animal facilities. This was a practical observation over several weeks that the attending neonatologists were aware of and suggested translation to the bedside. This know how was spread by worth of mouth only.

This study was approved by the Institutional Review Board (IRB) of the University of Texas Southwestern Medical School and conducted at Parkland Memorial Hospital, Dallas, Texas, USA. The IRB considered this pilot study to be a minimal-risk observational study of supervised standard care, and parental informed consent was waived.

Infants were chosen on a convenience basis during the day shift whenever a tube had to be replaced and a respiratory therapist and an attending were available. The intubations were performed prospectively alternating between NNETT and standard practice by second-year pediatric residents who were on their neonatal intensive care rotation. Standard practice (SP) consisted of using a bent wire trocar for the intubation and if chosen by the pediatric resident, with the help of a Magill forceps. 

The study was halted by the IRB when the superiority of intubation with the NNETT became evident.

## Results

**Figure 1:**
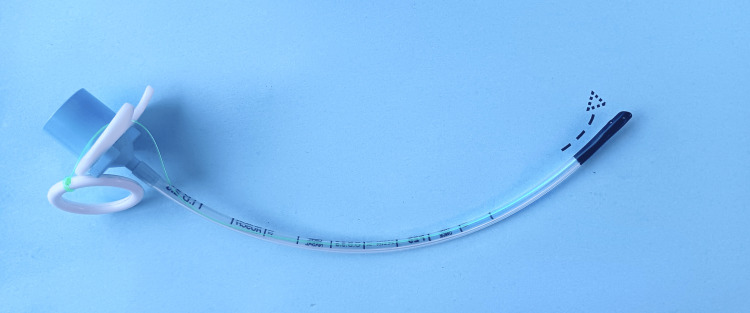
The new neonatal endotracheal tube (NNETT) A tension string (green) runs in a channel within the concave wall of the NNETT. It runs from the upper part of the tube's tip to the intubator’s finger ring (white). The directions of traction for the string are guided by a fixed or optionally detachable indented plate (white) that sits on the NNETT’s connector base (blue). According to hand size, the intubator places a finger, whether the third, fourth, or fifth into the white ring and, under finger control, pulls on the ring to lift the tip of the tube up (arrow) to slide it over the epiglottis and into the trachea.

**Figure 2:**
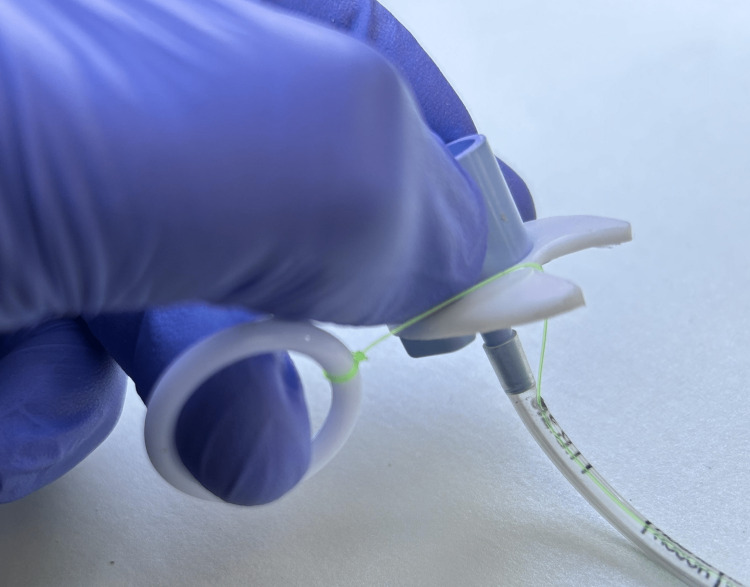
Hand and finger positions for intubation; shown is the left hand; either hand can be used

**Figure 3:**
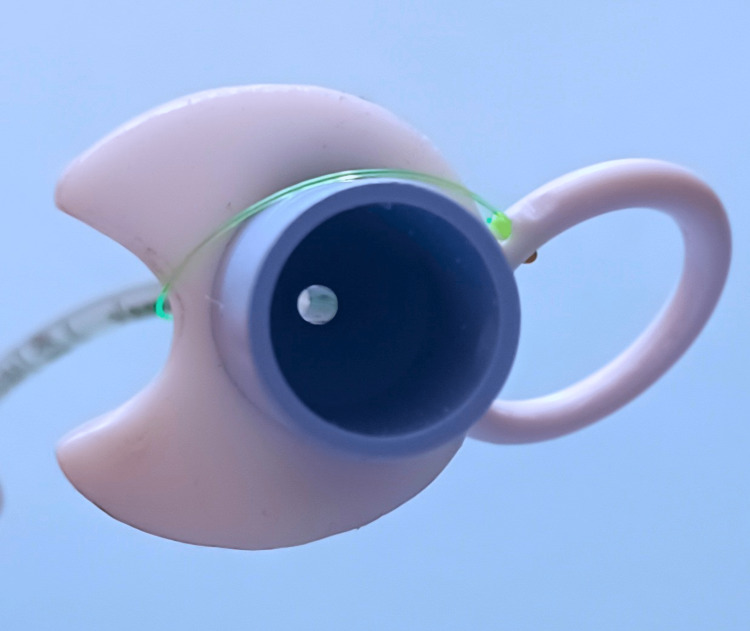
The endotracheal tube remains open throughout the process of intubation

The developed and new, optimized design features and their operation are presented in Figures 1-3. 

The first insertions using the NNETT design resulted in the immediate intubation of all six infants. By contrast, 13 insertions were needed to achieve the same number of six successful intubations by standard practice. The average intubation time with a mean of 36.6 sec and a median of 30 sec. was shorter with the new design compared with standard practice where it took a mean of 44.6 sec with a median of.45 sec. for intubation. With standard practice three attempts had to be aborted, and four resulted in the intubation of the esophagus.

Groups, observations, and intubation duration in seconds are shown and summarized in Tables 1-3.

**Table 1:** Size of endotracheal tubes (inner diameter in mm) used for each premature infant

Size of Endotracheal Tubes							Total
Premature Infant	#1	#2	#3	#4	#5	#6	6 premature infants (# assigned for this table)
Standard Practice	2.5	3.0	3.0	3.0	3.0	3.0	one 2.5 and five 3.0 mm inner diameter tubes
New Design	2.5	2.5	3.0	3.0	3.0	3.0	two 2.5 and four 3.0 mm inner diameter tubes

**Table 2:** Number of Insertions for premature infants including the final successful intubation

Number of Insertions							Total
Premature infant	#1	#2	#3	#4	#5	#6	6 premature infants (# assigned for this table)
Standard Practice	1	1	2	2	3	4	13 insertions leading to 6 successful intubations
New Design	1	1	1	1	1	1	6 insertions leading to 6 successful Intubations

**Table 3:** Adverse events in premature infants and the nature of the adverse events 0 = no adverse event; A = intubation aborted; E  = esophageal intubation; sec = duration of adverse event in seconds

Adverse events						
Premature Infants, # assigned for this table	#1	#2	#3	#4	#5	#6
Standard Practice - adverse event 1	0	0	E: 65 sec	A: 80 sec	E: 65 sec	A: 44 sec
Standard Practice - adverse event 2	0	0	0	0	E: 32 sec	A: 22 sec
Standard Practice - adverse event 3	0	0	0	0	0	E: 65 sec
New Design	0	0	0	0	0	0

**Table 4:** Time measured in seconds for successful intubations

Time in seconds (sec) it took for final intubation							
Premature Infant	#1	#2	#3	#4	#5	#6	6 Premature Infants (#assigned for this table)
Standard Practice	28 sec	34 sec	40 sec	45 sec	50 sec	71 sec	Mean: 44.6 sec, Median: 45 sec
New Design	15 sec	20 sec	30 sec	45 sec	68 sec	intubated by attending, no time recorded	Mean: 36.6 sec, Median: 30 sec

## Discussion

Training and maintaining proficiency in neonatal intubation has become more challenging when the main teaching opportunity of the past, the mandatory suctioning and immediate intubation of neonates born with meconium in their amniotic fluid, was discontinued as a procedure without clear evidence of benefit [12]. 

Fewer intubations are performed due to the advances in neonatal resuscitation with continued positive airway pressure (CPAP) [13], and the use of less invasive surfactant administration (LISA) which is performed through a thin temporary catheter, obviating the need for intubation. LISA is deemed to be superior to the administration of surfactant through an endotracheal tube [14], which may stay in place for several days.

Smaller laryngeal mask airways (LMA) for premature infants may become available. These keep the airway open over a limited amount of time and their placement does not require a laryngoscope, video laryngoscope, or intubation skills [15].

Adaptations of medical instruments to the anatomy of the trachea have been realized over the course of human history. Advances in airway instrumentation occurred from the earliest description around 2000 BC until the middle of the twentieth century [16]. 

Recent examples in adult practice are the Ballard laryngoscopes which have a special curved tip, allowing for so-called blind intubation [17], and the Endotrol^TM^ tube (Medtronic Ltd., Watford, UK) where the tip of the endotracheal tube is pulled up by a string, similar to the mechanism developed for this study. Martay and Hunter presented the Seattle Endotracheal Tube Series [18], which refers to cuffed endotracheal tubes that are based on the Endotrol^TM^ design. They incorporate a thinned wall on the upper part of the tip of the endotracheal tube that allows for the selective bending of a rigid tube tip. However, the pull string of the Endotol^TM^ tube must be pulled in the direction of the connector to avoid a rotation of the tube, and from Martay and Hunter's depictions [18], it appears that the tube is also briefly occluded while the intubator is inserting the tube.

All of these intubation-facilitating approaches were developed for adult patients and apart from the commercial return-on-investment challenge for neonatal devices [19], such endotracheal tubes would be hard to adapt to the smaller and easily traumatized airway of a preterm infant.

Several fiber optic trocars and visualization camera laryngoscope systems [20] are on the market to facilitate endotracheal intubation. Fiberoptic trocars still occlude the tube, and similar to video laryngoscopes, they are still costly to acquire and will therefore not always be available in resource-constrained environments when an emergency delivery of a preterm infant occurs. 

The mechanical wires and trocars that are currently used to curb the tube to facilitate intubation have not seen any substantial improvements. They can traumatize tissues, and wire trocars may perforate the trachea (or, by misplacement, the esophagus) when they are not adjusted to the length of the tube. They also impede airflow through the tube during intubation. 

These situations called for innovations in the intubation of preterm infants, of which a simple, economical, and very effective one is offered here.

## Conclusions

The design features presented and tested here for the intubation of preterm infants allow for patency to be maintained over the course of intubation and for the intubator to advance the tube without trauma and under full vision.

The new design, patent-pending, outperformed conventional endotracheal intubes in preterm infants. The prospectively monitored comparative observations were sufficient for the Institutional Review Board of the University of Texas Southwestern Medical School in Dallas to halt the reported study because success was self-evident. However, that success cannot benefit future infants without commercialization. This device remains an orphan product.
